# A Facility-based Family Support Intervention to Improve Treatment Outcomes for Adolescents on Antiretroviral Therapy in the Cape Metropole, South Africa

**DOI:** 10.1177/23259582211059289

**Published:** 2021-11-25

**Authors:** Zaida Orth, Brian van Wyk

**Affiliations:** 1 School of Public Health, 56390 University of the Western Cape, Bellville, South Africa

**Keywords:** photovoice, adolescents living with HIV, adherence, family club

## Abstract

Adolescents living with human immunodeficiency virus (HIV) (ALHIV) globally, report worse treatment outcomes compared to adults and children on antiretroviral therapy (ART). We conducted a photovoice study with eighteen ALHIV to explore experiences and challenges of being on ART, and individual interviews with 5 health workers to describe the challenges in treating ALHIV. The facility implemented the *Family club* intervention to facilitate caregivers (parent/guardians) supporting ALHIV on treatment. The health workers revealed that “*disclosing HIV status*” to children was the biggest challenge for caregivers and health workers. Participating ALHIV reported that *family support* and having a *positive mentality* were instrumental for continued treatment adherence. However, *disclosure* of HIV status to friends remained a challenge due to pervasive community stigma. *Treatment fatigue* and *side*-*effects* were also barriers to adherence. Family support was instrumental in facilitating adherence support for ALHIV. However, this (intervention) should include peer support to improve positive mental well-being in ALHIV.

## Introduction

The increased availability of antiretroviral therapy (ART) combined with the scaling up of health care services and programmes aimed at supporting adherence to treatment has increased the life expectancy of people living with human immunodeficiency virus (HIV) (PLHIV), including perinatally infected children who are now surviving into adolescence.^1,2^ The increased survival rates of perinatally infected children along with the growing number of behaviorally infected adolescents have resulted in adolescents being identified as the fastest-growing population of PLHIV. As such, adolescents have been identified as one of the key populations in the fight against HIV. According to UNAIDS 2019, the global population of adolescents living with HIV (ALHIV) was estimated at 1.7 million.^3^ In South Africa the number of ALHIV was estimated at 360,000 (230,000-500,000), with 31,000 (8100-62,000) new infections reported in 2019.^3^

Globally, it is estimated that there has been a 45% increase in AIDS-related deaths among adolescents between 2005 and 2015.^4^ This is of great concern, as it stands in contrast to the decrease in AIDS-related deaths reported for all other age groups.^5^ The increase in AIDS-related deaths among ALHIV suggests lapses in treatment and successful engagement. Evidence suggests that this lapse in treatment may be associated with the type of care and the quality of support that ALHIV receive.^6^ Adolescence is a unique developmental period in which individuals experience significant physical, psychological and social changes.^7-9^ Our report from an exploratory study of ALHIV on ART in a low socioeconomic setting in Cape Town also revealed that; school versus health facility conflicts, negative household dynamics and finding the health facility as an unfriendly place, as reported barriers to adherence.^10^ Therefore, the World Health Organization (WHO) recommended that public health care facilities establish adolescent-friendly health services which include age-appropriate treatment programmes to provide psychosocial support, sexual and reproductive health services, and improve adherence and retention in care.^1^ However, poor treatment outcomes for ALHIV are also influenced by a variety of person and social factors such as treatment fatigue, delayed disclosure of HIV status, stigma, lack of social support, mental health challenges, substance use and transitioning from paediatric to mainstream care.^11,12^ Our review of psychosocial support interventions for improved adherence and retention in care for ALHIV indicate promising results from 2 interventions with family-centered services namely the family day clinic (FDC) and the VUKA family programme.^13^ Both of these interventions involved multiple components including counseling services with ALHIV and their family members and health education workshops to improve retention in care and adherence among ALHIV.^13^ Results from both interventions show a significant increase in adherence among ALHIV compared to the control groups. However, reports from the FDC showed no effect on retention in care between the control group and the intervention group.^14^ Our analysis of a cohort of ALHIV newly enrolled on ART in the Western Cape Metropole showed that retention in care declined by 60% over 2-year period^15^ and that only 25% of the initial cohort attained viral load (VL) suppression.^16^

In the current paper, we describe the implementation and experiences of a facility-based family support intervention (family clinic) in a public primary health care facility in the Cape Metropole in South Africa to address challenges in adherence and treatment outcomes for ALHIV on ART.

## Methods

### Study Design

The photovoice study is part of a larger research project that aimed to improve treatment outcomes—adherence, retention in care and viral suppression—for ALHIV who were receiving ART at public health care facilities in the Cape Metropole in South Africa. Photovoice is described as a process by which participants can “identify, represent, and enhance their community through a specific photographic technique.”^17,18^ We chose photovoice methods in this study to engage ALHIV in data collection in a way that allowed them to “decide” what is important to them relative to their experiences and challenges of being on ART. The photovoice sessions aimed to give voice to ALHIV, while also allowing peer interactions, with no interference by the researchers.

We triangulated the findings from the photovoice sessions, with individual interviews with key health care workers (HCWs), to situate the ALHIV's experiences within the health services context.

### Context

The public health care facility has been in operation since December 2014 and offers health care services to a catchment population of 90,000 in predominantly low socioeconomic income communities. The clinic provides 24-h emergency services and day services from 07:30 am to 4 pm and includes a comprehensive package of care including chronic care; child health; emergency services; infectious diseases treatment; obstetrics and gynecology; oral health; pharmacy; rehabilitation services which includes occupational therapy; physiotherapy and speech therapy; digital radiography and women's health. Additionally, the clinic works in collaboration with nongovernmental organizations to provide mobile HIV services, especially HIV testing and counseling, voluntary medical male circumcision, and sexual reproductive health education. However, at the time of data collection, these services were on hold due to an interruption in government funding.

### Sampling and Participants

During the initial phase of the study, the research team made contact with key HCWs at the clinic and informed them of the purpose of the study. Due to the sensitive nature of the study, the HCWs agreed to assist the research team with the identification of eligible participants. Our inclusion criteria were as follows:

- adolescents between the ages of 10 and 19 years at the time of the study;
- with a confirmed positive HIV diagnosis, and receiving ART at the clinic; and
- must have been disclosed to and aware of their HIV-positive status.

A nurse, qualified in nurse-initiation and management of ART (NIMART), assisted with the participant identification and initial recruitment. The researcher (ZO) met with the head nurse and potential participants to discuss the study and provided them with information sheets and assent/consent forms. Those who agreed to participate were asked to return signed consent forms by both themselves and their parent or guardian, and provide the contact telephone numbers to arrange the group sessions.

Our realized sample consisted of 18 adolescents. We conducted 6 groups—3 with older adolescent female groups of 3 to 4 participants (15-17 years) each; 2 with male groups of 3 participants (15-17 years) in each group; and 1 group consisting of 2 younger adolescent girls (both aged 10 years).

Additionally, the head nurse introduced us to key HCWs who had experience working with adolescents on ART. After receiving their written consent, we conducted individual interviews with 5 HWCs to explore how they perceived treating ALHIV, and the challenges they experienced.

### Procedure for Photovoice Sessions

In the first session, participants returned their signed consent forms and received a cell phone camera with which they would use to take their pictures. During this session, the research team provided more details and information regarding the study and showed them how to take pictures with the phone. Participants were instructed to take at least 5 pictures that tell their story about their experiences with ART. We explained that we were interested in understanding the challenges they may experience, as well as their motivation for taking their treatment. Additionally, the researchers discussed safety precautions with the participants and cautioned them against using the phones in areas where they may be subject to theft or harm. The first session also allowed us to introduce the participants to each other as a way to facilitate group rapport. We arranged with the participants to return to the clinic a week later to take part in the focus group discussion.

During the second session, the participants were given the opportunity to share their pictures on a projector screen which prompted discussion in the group. Each session was facilitated by 2 members of the research team. Even though the participants conversed in English, we were aware that for most of them, English was their second language with isiXhosa being their home language. As such, we arranged for an isiXhosa-speaking counselor or research assistant to attend sessions to allow participants to speak in their home language whenever they prefer to do so.

During the data collection phase, the researcher approached key HCWs to request individual interviews. Those who agreed to participate signed a consent form and arranged a time and date for the interview. The information from these interviews provided further context, which helped us to facilitate the focus groups.

### Data Analysis

All interviews were audio-recorded, transcribed verbatim and translated where necessary. Photos were inserted into the transcripts, where these were referred to during photovoice discussion. All transcripts with photos were uploaded on Atlas.ti and subjected to thematic analysis.

### Ethical Statement

Ethical approval to conduct this study was granted by the University of the Western Cape Biomedical Research Ethics Committee (BM18/3/7) and the Western Cape Health Research Committee (WC_201807_003). As previously mentioned, all the participants included in this study were provided with information about the study in a language they understood and signed the informed consent sheets before participating in the study. Additionally, adolescents younger than 18 were required to obtain consent from their parent/guardian before participating in the study. To protect the anonymity and confidentiality of the participants, all identifying information has been changed and participants were given pseudonyms.

## Findings

Our findings highlight the health facility's main interventions to deal with the challenges of maintaining consistently high levels of adherence and engagement in care among ALHIV on ART, namely the “Family club” and the “Risk of Treatment Failure” (ROTF) clinic. Other themes reflect other challenges experienced by health workers as well as ALHIV experiences of *Family Club, ROTF Clinic, Disclosure, Motivation and Challenges to Treatment Adherence* and *Family Support*.

### The Family Club

The clinic has established a *family club,* which was championed by the head nurse/HAST (HIV/AIDS, STI's and Tuberculosis.)manager. The family club consists of 25 to 30 HIV + family members who were clinically stable—meaning that they have been on ART for at least 6 months and are virally suppressed. The family club supports patient adherence by providing patient-friendly access to ART. The family club sessions occur once every second month and last 30 min. During these sessions, the head nurse or another trained NIMART nurse noted attendance and briefly addressed the members in the family club to discuss any problems they may have experienced since the last meeting. Following this, the medication is distributed and a date for the next meeting is confirmed. At least 1 family member needs to be present to collect the medication for the rest of the family. This is beneficial as the parent or child would not have to miss work or school to collect their medication, as explained by the adherence counselor below:

It works and it is nice, because one person that is gonna be off at work or at school, ne? Sometimes the schools are closed like this and the child will come and pick up the medication for all of them, or else…the child will come after school to pick up the medication. They will find the medication in the same place—Adherence counselor (Female)

However, all family members are expected to be present on the day when they need to have their blood samples taken for VL monitoring—which occurs at 4 and 12 months after ART initiation, and then annually thereafter.^19^

The pediatric outpatient clinic offers a similar service to parents of stable HIV + children. This may be useful for parents of younger children who do not yet qualify for placement in the family club.

Uhm, there is the family day club with Head NIMART Nurse, but it's also here by us in OPDs side cause sometimes the parents want them in school as well. So then they will come, but they will come like every—say they must come in two months’ time then the parents will come in 2 months and then the next time they have to come because their weight can change, the medication can change—Doctor 1 (Female)

While the family club and counseling services are shown to provide instrumental support and easy access to treatment, there is still a recognized need to provide more social support for ALHIV in the clinic.

They need more social support, yes. Which we…I don't feel we do enough. We try, but it's not enough—Head NIMART Nurse (Female)

### Risk of Treatment Failure Clinic

The ROTF intervention was first piloted by Médecins Sans Frontières in 2012 before being adopted by the Western Cape Department of Health in 2015 to manage all patients who are failing or at risk of failing ART.^20^ ROTF is designed to provide integrated adherence and clinical management for support patients whose VLs are not supressed (>400 copies/mL) regardless of their treatment regimen. Patients who experience a single unsuppressed VL are enrolled in a support group, while patients presenting with 2 consecutive unsuppressed VLs are given more intensive counseling. These counseling sessions include personalised educational activities which seek to address the patient's reasons for defaulting on their treatment. Adherence is managed by monitoring the patients’ VLs and switching to second-line ART regimens as per the national guidelines. As the quote below shows, patients in the ROTF clinic are afforded more time with the HCWs and counselors to learn specific and targeted tools which may help them to overcome barriers to treatment adherence and engagement in care.

Once you’ve got someone that's defaulting, obviously your viral load is gonna be high. So, you sitting with like a unsuppressed viral load. So, they go into ROTF. So now in that ROTF they have been doing very well because I mean, there they get the chance to see other patients’ perspectives and what their struggles are. “Okay, I’ve been doing something wrong as much as I don't want to admit it.” And they go home and change that behaviour. And they open up to the counsellors and they open up in that session, that one-on-one, where … We individualise with them and they get that specific cover that half an hour, whatever and we sit with you and we like “okay we gonna get to the bottom of this and give you like … tools to help you to be compliant.” So, there is time for that.—Doctor 2 (Female)

In one of the photovoice discussion groups, Grace (Girl, 16) described how she received support and motivation from the head nurse to attend the ROTF clinic, and in this process started adhering to her treatment again.

So then that's when my viral load was like so high, it was like 4000 or something. And then Sister made me see the point of taking my pills and slow by slow I started taking it again. Because I always blamed my parents for my … situation—Grace (Girl, 16)

However, Grace (Girl, 16) reported that she struggled to relate and open up in the ROTF group because the group mainly consisted of older adults. This was in contrast to her experience of the photovoice discussions where she was more comfortable opening up to her peers.

When I was Sister, it was the RTX something. It was only old people there, you know. And you hear them talking a lot … like what am I doing here? So I feel much more comfortable and more happy now.—Grace (Girl, 16)

### Disclosure of HIV status

Most of the ALHIV who participated in this study were infected from birth and recalled taking the medication from a young age. However, they were only disclosed to when they were older (between the ages of 12 and 13 years). The participating adolescents reflected on having different reactions to being disclosed to from initial denial (like David below), to anger and withdrawal (like Emma and Aaliyah, who had siblings who are HIV negative) and blamed their parents (for failing to take action to protect them from infection).

I said it can't be true.—David (Boy, 15)

When I first found out I was…12. Yeah, I didn't speak to my mother for I think like … a week—Emma (Girl, 17)

Because I have a younger brother and he wasn't breastfed, so I was like ‘why him? Why did you have to breast feed me?’—Emma (Girl, 17)

It was 2016 … I was 13. My aunt sent me to the clinic—so I found out there. So I was kind of … angry … and I blamed my parents … and I kept asking myself why because my younger brother doesn't have it and my older sister … so why me?—Aaliyah (Girl, 16)

However, others like Faith (Girl, 16) remembered taking the medication since she was young but reported that she did not know what the medication was for. She explained that she was fine with taking the medication once she understood the reason behind it.

Yeah, but I took my medication since I was young, but then I didn't know. I was still young; so I didn't understand why I am taking this medication. But like [unclear] they did do…like, I found out from the doctor that I am … [HIV] positive. So I started asking myself so is that the reason why I have been taking my pills. And then they said it's fine as long as I keep taking my medication. So I was fine with that.—Faith (Girl, 16)

The decision to disclose to the child his/her HIV status is usually discussed between the parent/guardian and the doctor/nurse. Once the child has been disclosed to, they will refer them for counseling to the adherence counselor.

We not telling them that you know, “why I am taking this medication”—no it's doctors that doing that, then they come to us once they know why they are taking the medication. And then just to emphasise to them why it is important to them to use their medication, what can happen if they can stop their medication—all those things”—Adherence counselor (Female)

In counseling the adolescent is asked about their HIV status and being on ART, they (counselors) seek to help the child understand why the medication (and adherence) is important and to focus on the positive(s)—that they are not sick—and do all that they can to remain not sick. Every effort is made to divert the adolescent away from negative thinking about how and why they were infected, and negative emotions towards their parents or their situation.

I always say when I am speaking to them—for even if it's an adult in fact—for us to check when did you get HIV, how did you get? It's not gonna work for us. So let us put aside that “why do I take medication?,” “when did I get? how?,” because it's gonna make you stuck. You, you not gonna continue with your life if you want to know why to me? Why do I have HIV? Why me? So, I am always saying to them, once you find out—it's nice to find out, while you work on yourself, you are not sick, you just need to follow the procedures of the hospital—no one can know you are on treatment.—Adherence counselor (Female)

According to the HCWs, delays in disclosure to adolescents about their HIV status often leads to poor adherence and defaulting on treatment. The head nurse described cases where a adolescents were disclosed to at an older age, when defaulting on treatment occurred, and how this disclosure affected their mental health. While there are counseling services available at the facility it is difficult to follow up with adolescents who have depressive symptoms due to the limited capacity of the community workers and the health care staff

Uhm … I had one last week. It's actually … that is a big problem because, when the girl or the boy becomes 13/14 years old, the parents don't tell them what tablets they are taking. They give the children tablets because they just uhm, they make sure that the child don't get sick because there is flu around and everything … the children don't know that they are HIV+. When they find out, it's a big thing. They go into a depression, they come to the hospital, their viral loads are high—I had one last week that said she's fed up now of taking this tablet—she didn't know. So, the social worker are involved, I am involved, the counsellors—we counsel them and we keep a close eye on them It's very difficult to keep a close eye on everybody because … they live in Du Noon, and there's a lot of burns and protests and things going on here. A 1000 houses were burned out two weeks ago—Head NIMART Nurse (Female)

Only 2 participants found out about their HIV status through voluntary HIV testing. Below are the accounts of Zola (Boy, 16) and Amy (Girl, 17).

Yes when I got tested and saw the results. Everything in me… it felt like life just stopped. Then I found out from the lady who was assisting me that there are others living with HIV and that I could get support from a group. She really encouraged me, and gave me the information that I needed. I told myself I can't change my status, I have what I have but I also have to continue living my life.—Zola (Boy, 16)

And then I decided to just go there and…test if maybe I am HIV + or not uhm…I tested and then was surprised it came up positive. I was like ‘How?’ how? how? I was asking the same questions like Amahle uhm…and then I remembered okay…it's fine, I, I did not even cry—to my surprise, I did not cry because…I, I, I kept on telling myself that's okay, it's life, maybe it's a step you have to go through… And then okay, I accepted it and then, when I see a tree…a tree is strong and it, it reminded me okay if I take my pills I’ll forever be strong—Amy (Girl, 17).

### Motivation to Adhere to Treatment

**Figure 1.**
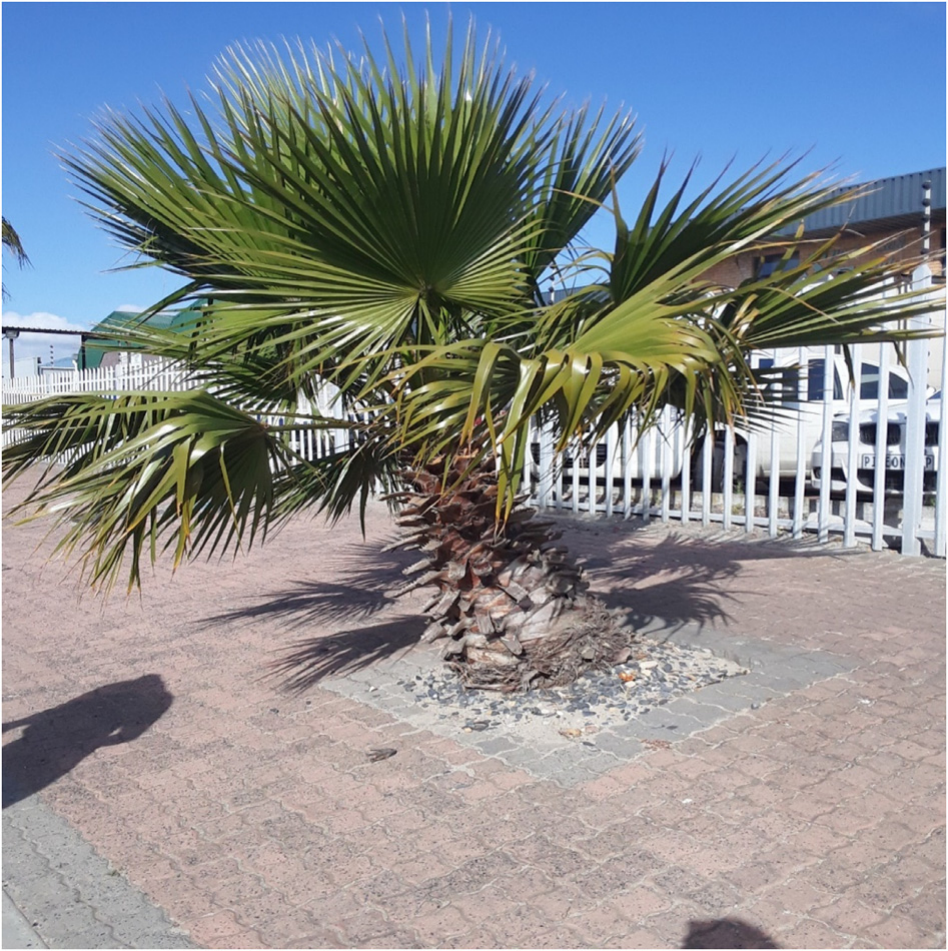
Adam (Boy, 14)—tree.

Most participants reported being adherent to treatment. During the photovoice discussions, some participants showed pictures of flowers, trees and the ocean to express their positivity about living with HIV and how they stay motivated to adhere to treatment. Adam (Boy, 14) showed a picture of a tree and explained that as the tree, under the right conditions, he will also continue to grow and thrive when he adheres to his treatment.

the tree here tells me that if a human being continues to take his medication, he will grow up and feel free, and the trees have a good life and … and have a healthy, happy life, and the tree here is strong. That shows that when a human being … takes care of his or her body … will be strong, fresh, energetic … and feel good for their life—Adam (Boy, 14) (Figure 1)

Participants also displayed good knowledge on the importance of treatment adherence and understood the consequences of not taking their treatment, which motivated them to adhere.

Well, my mother encourages me, I have to take my pills because that's the only way my counts will be undetectable? And then I won't be able to uhm, infect other people—Emma (Girl, 17)

I feel happy [about taking the treatment] because I want to be…I want to be healthy—Precious (Girl, 10)

For Zola (Boy, 16) looking healthy was also very important, as this will help them to conceal his status. This motivated him to be adherent to treatment.

I like taking my treatment because it helps me. It makes me look and seem like I don't have what I have. Like, people can't see that I have it. It makes me feel healthy and people don't know that I have it—Zola (Boy, 16)

Some participants’ motivation was based on fear and dread. For example, Jabu (Male, 17) states, he continued to take his medication because he did not want to die. Amahle (Girl, 17) took a picture of her shadow and explained that she has accepted that the virus is a constant fixture in her life and that she carries her pills in her pocket all the time.

**Figure 2.**
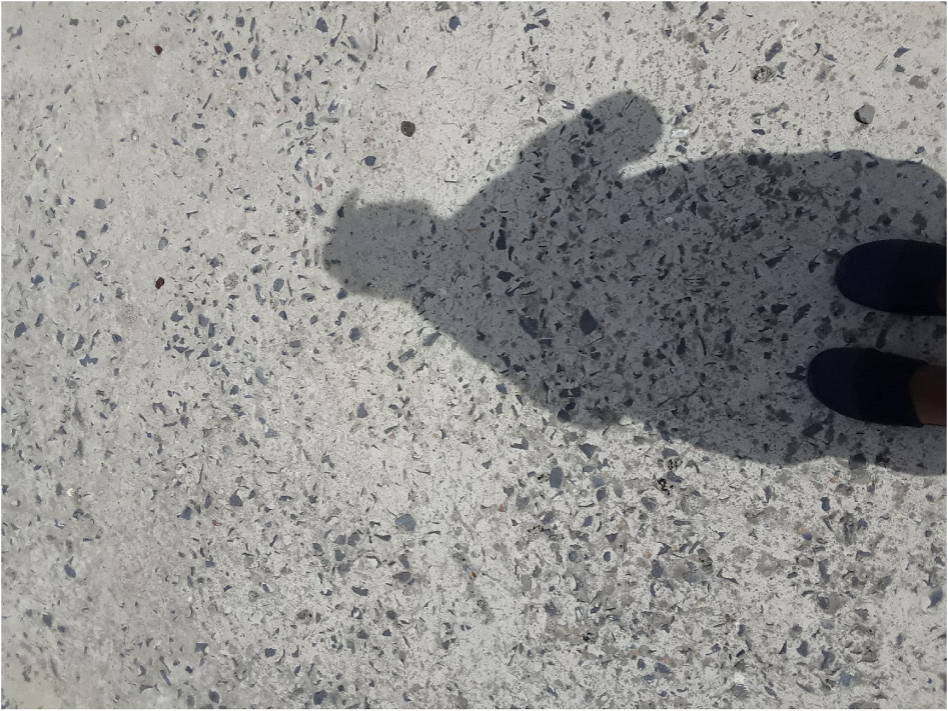
Amahle (girl, 17)—human immunodeficiency virus (HIV) is my shadow.

“Guys that's me, that's my shadow. That's my friend … I have to accept it … my friend, wherever that I’m walking with my purse, in my pocketI have to because my pills, that's my—if I stop drinking it then that's the end of me. That's my friend—my virus … all the time she was—okay, all the time I must go and … all the time”—Amahle (Girl, 17) (Figure 2)

Many participants reported that their motivation for taking their treatment and coping with their HIV diagnosis comes from a love for and desire to take care of a family member—mother or child—as described below by Kaya (Girl, 16) and Sarah (Girl, 17)

It's a tattoo. It was on Yara's instagram. Yeah, I guess she posted on her phone; so I take the picture and then I take a screenshot. So, at that time I was going through a rough time. It was the same thing of like “why should I take my pills?” I really don't like taking pictures, but for some reason… like I love my mom and I need to pick a prompt for this. So, this “thingy” just makes me believe everything will be fine, so I have to look after myself. So yeah, I took it. It gives me courage.—Kaya (Girl, 16)

**Figure 3.**
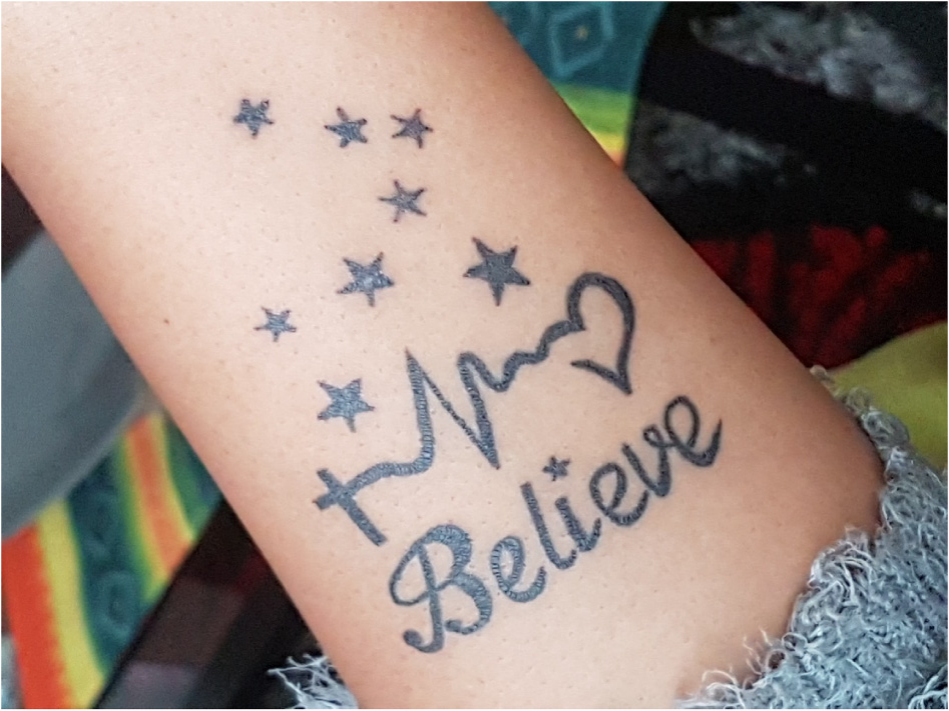
Kaya (girl, 16)—tattoo.

“My mother is the greatest born creation. Like she does everything for me so, actually one day I have to do the same thing for her when she's old.”—Kaya (Girl, 16) (Figure 3)

Similarly, Sarah explained that like the picture of the car that was moving, she also feel motivated to move forward in life, and be in a position to take care of her son.

**Figure 4.**
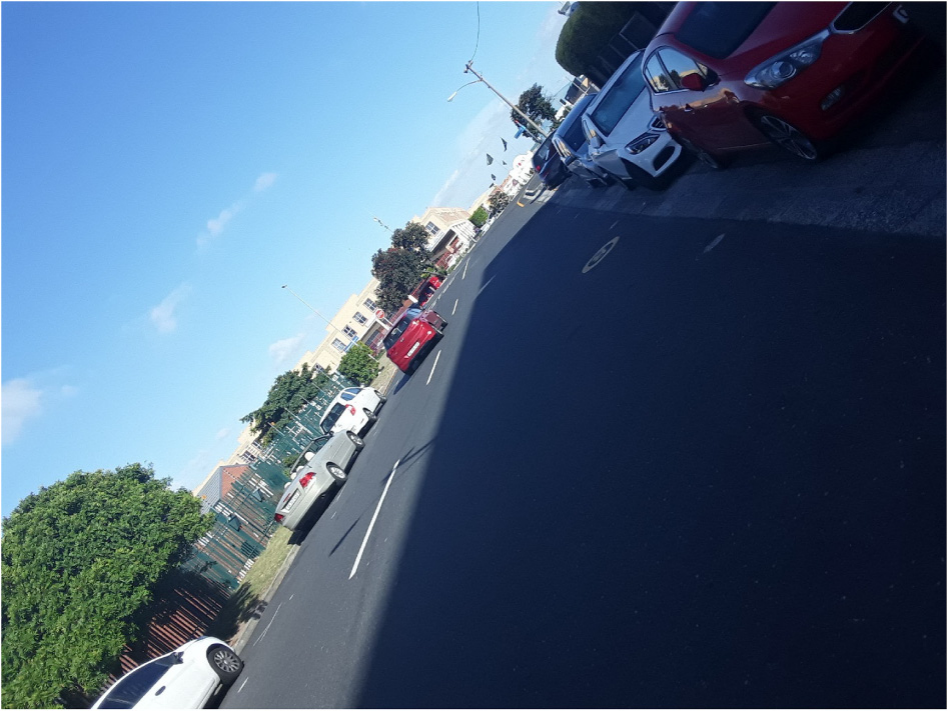
Sarah (girl, 17)—moving forward in life.

The second one is the picture of … okay a car. Okay, I took picture of a car and the car is moving. It means life goes on after all. I found out that I’m HIV+ … I am still a student and, I am a mother on top of that…my son needs me, I need to move forward and leave everything behind. Yes, I know that I can never forget the fact that I am HIV+ … but it's the thing that I have to live with it for the rest of my life, because if I stop taking my pills, I’m gonna die or leave my son behind and that's not my wish”—Sarah (Girl, 17) (Figure 4)

Grace also, was motivated to live (and take her treatment) by love for her younger sister, and this is stronger than the struggles that she is experiencing with her HIV diagnosis.

You know she gave me hope, like she's so young and I’m sure she doesn't want to lose her sister. And I still want to see her grow and be beautiful and all this stuff.—Grace (Girl, 16)

Grace went further to describe how the support she received from the Head NIMART nurse was instrumental in getting her to recommit to her HIV treatment.

She's very vibrant. She's very supportive, I can say. She's like: “It's life but, you still have a long way to go. Don't make anyone decide who you are. Don't make this disease decide who you are, because you’re not that” —Grace (Girl, 16)

### Challenges With Medication Adherence

Even though particpants reported that for the most part they do not have difficulties in taking their treatment, there were times when they found it challenging to stay motivated.

So that is my pills (showing a picture that she took of her pills), so sometimes…sometimes I don't wanna take the pills it gets stuck in my throat -Emma (Girl, 17)

Amahle also showed a picture of a closet she used to hide in to avoid taking her treatment when she was younger. For both Amahle and Emma, the resistance to taking their pills was based on the discomfort of the pills that are hard to swallow and becoming nauseous because of the bad taste it left in their mouths.

**Figure 5.**
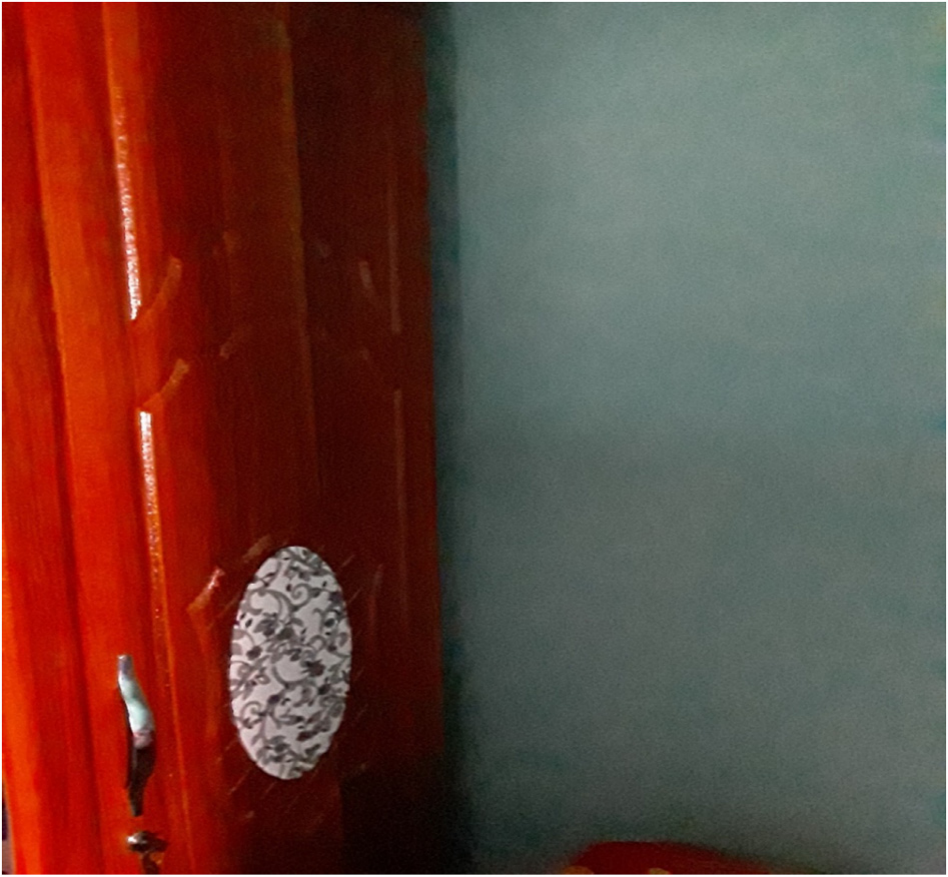
Amahle (girl, 17)—hiding in the closet.

Okay, after the syrup then, when I was in the Eastern Cape, when I was 10 years old, I moved from the syrup to the pills, but the pills—okay, why I was doing hide and seek because at first, I want to vomit after drinking, there's that aftertaste—I tell myself to be strong but after—after drinking I take a sweet—Amahle (Girl, 17) (Figure 5)

However, Amahle's admission regarding her struggle to take the treatment prompted conversation from the other participants who shared their struggles with dealing with the physical effects of the tablets and shared some tips to overcome it.

### Family Support

Most participants relied on their parent(s) or a family member to remind them to take their tablets. In Amahle (Girl, 17) and Faith's (Girl, 16) cases, they were the only HIV+ members in their household. Even so, they could still rely on their family members to remind them and support their treatment adherence.

No—oh my parents and my sisters always remind me because I sometimes forgot and when straight to bed, after that, oh my God!—Amahle (Girl, 17)

Yeah always, or when I forget my mom always reminds me and my cousins, so I have them—Faith (Girl, 16)

In households with more than 1 HIV positive family member, as in the case of Amy (Girl, 17) and Bongi (Boy, 15) they held each other accountable for taking their medication daily.

If I forget to take pills, she reminds me, and if she forgets, I remind her. We support each other—Amy (Girl, 17)

Uhm, what makes is easy, it is that I…there is also my mother on my side that also has HIV, that also tell me when is the time, and we could also support each other—Bongi (Boy, 15)

However, while family members are seen as a source of structural support, some participants revealed that there are limitations to turning to family for emotional support. Kaya explained that she does not discuss her problems with her mother because she does not want to be a source of worry for her.

Sometimes when I stay in my room then she asks me what's wrong and I don't feel comfortable because she gets so worried quickly. So, I don't want to stress her out so I just keep quiet. But then I hate seeing her stressed. Like yah she can smile but for me I can notice that she's not ok.—Kaya (Girl, 16)

On the other hand, Amahle (Girl, 17) revealed that while her family supported her with treatment adherence, she has trouble opening up to her family because she does not trust them with her emotional problems.

I don't trust anyone. To my sister sometimes…okay, to my other sister, I’m open to her, but not everything. And my mother used to ask me ‘you okay?’ so yeah. Because I’m just—I’m not a talking person, but there's times where I want just, if I talk I…if I talk to someone I just get better and no ‘why, why, why, why?’—Amahle (Girl, 17)

During the interview, Thandi (Girl, 15) listened to the other participants describing the support they received from their family members and started crying as she reflected on her own lack of support at home. While Thandi (Girl, 15) does have a good relationship with her cousin and aunt, she described that her relationship with her father is strained.

Uhm, how can I say? It's just that he doesn't give me enough support that I need. I don't feel like … I don't feel like he cares and … because we don't live happy”—Thandi (Girl, 15)

### Disclosure of HIV status to Friends

Some of the participants took pictures of their friends and discussed their friendships as a source of emotional support and happiness, which help them to cope with life stressors.

Like, I move on because when I arrive at school, I’ll be happy so I, my friends are the best thing to me, so yah.—Lerato (Girl, 15)

So uhm, that is where I like going when I am sad and all that. So, what makes me happy is uhm, going out with friends and sometimes family. I also love food [laughter]—Emma (Girl,17)

However, this prompted conversations around participant's willingness to disclose their status to their friends and the people they are close to. As demonstrated in the quotes below, disclosing and sharing one's status is a complicated process. As the quotes from Emma suggest, even though she is close with her friends, she understands that these relationships may be temporary and could change as they complete school and go their separate ways. However, Emma also mentioned that she is reluctant to disclose as she believes her friend would not be comfortable with the news about her HIV status.

I share everything but not my … sickness—Emma (Girl,17)

Cause I wouldn't want to tell somebody and then in the future they’re not part of my life—Emma (Girl,17)

I feel like she would be uncomfortable—Emma (Girl,17)

For others like David (Boy, 15), it is about getting the timing of disclosure of HIV status to his friend right. Whereas he wants to share this information with his best friend, he is also aware that the disclosure may affect their friendship negatively if done too soon—before both parties are ready and able to deal with this.

No. I would tell my friend. There is a friend that I have and I would tell them about it because I trust him. But he must be ready first. David (Boy, 15)

In stark contrast, Grace related that her friends know about her HIV status and support her by reminding her to take her treatment.

It's not like I’m meant to die. So, I have two best friends and they know about this pill. So, like they motivate me every day to get well. At like 6 o’clock the send me a message “take your pills”. I’m just like, what's the point anyways?—Grace (Girl, 16)

## Discussion

The findings of our study demonstrate the commitment of the HCWs in this public primary health care facility to implement best-practice interventions to support ALHIV in their treatment. Previous studies have shown that enhanced adherence counseling interventions, such as the ROTF clinic, have a high probability of success in re-engaging patients who have defaulted on ART to be adherent again.^19,21–23^

Similarly, it has been shown that psychosocial support interventions such as “family clubs” that engage families in supporting children and adolescents on treatment are successful in removing or reducing barriers to adherence in addition to providing psychosocial support. Our findings suggest that family support interventions had high acceptability among ALHIV and their family members because in many cases, there were more than one family member on ART. However, Grimsrud et al^19^ suggest that it is necessary to create a balance between engaging the family to support ALHIV and fostering a sense of independence and self-management as ALHIV transition to the adult treatment programme. They suggest that the abovementioned process of transition to adult care should be accompanied by transistioning adolescents from the Family Club to a Teen Club to facilitate adolescent-specific peer support.

By definition, the Family Club is geared for families, where at least 1 parent and 1 child is HIV-positive and on ART, older adolescents who were behaviorally infected are typically excluded from this model of care.

Similarly, ALHIV can only participate in the ROTF if their VLs are not suppressed. The account of the ALHIV in our study in the ROTF clinic narrates that this is a lonely journey for an adolescent, as they mostly interacted with adults—which left them unable to relate and not feel comfortable to share their challenges with treatment adherence. Research on Teen Clubs has reported that these clubs are mostly effective in improving adherence and motivating retention in care among ALHIV. Additionally, the growth in Teen Club memberships globally suggests a high level of acceptability among ALHIV.^24,25^ It is suggestive that Teen Clubs may be a useful platform for delivering enhanced adherence counseling for ALHIV who are ROTF.

Our study confirmed that disclosure of the child's HIV status is a challenge for all adults involved. However, previous research has consensus about the benefits associated with HIV disclosure, in improving mental health and well-being, adherence and treatment outcomes.^2,26^ In a review of factors influencing adherence among ALHIV, Ammon et al^27^ identified disclosure as the main barrier to adherence after stigma, because the child/adolescent did not know the reason for taking the medication. The WHO and the American Academy of Pediatrics have set out guidelines to support age-appropriate disclosure of HIV status to children to achieve better treatment outcomes and to improve psychological adjustment.^28^ WHO recommend that (partial) disclosure to the child should begin at age 6 years, with full disclosure completed by 12 years of age.^29^ This resonants with our study findings where most of the perinatally infected participants were disclosed to as early adolescents (12-13 years). As reflected in Nichols et al's^26^ synthesis of 14 quantitative studies reporting on the effects of disclosure on adherence—where 5 showed no association, 5 showed a benefit to disclosure and 4 showed a negative impact—the experiences reported by participants in our study were varied. The negative impact of disclosure on adherence was precipitated in denial of HIV status, followed by anger and resentment towards parents, and refusal to take their medication for specified periods.^26^ It has been reported that disclosure is followed by an increase of depressive symptoms which is associated with a decreased desire to form and maintain healthy adherence habits and loss of hope for the future.

Similar to previous studies, we found that the HWCs believed that early disclosure is important as they associated with adherence problems among ALHIV.^30^

Our study indicated that delayed disclosure was attributed to the readiness of the parents/guardians rather than the readiness of the child/adolescent. To address this issue, HCWs in the participating facility followed a specified guideline to prepare parents/guardians for disclosure. The majority of ALHIV in our study reported that they were able to get over the initial shock of disclosure and come to terms with their diagnosis and focused on their health and their future aspirations. This may be attributed to the supportive environment created by the Family Club and the meaningful participation of HCWs in the disclosure process.^23^ Additionally, upon being disclosed to, ALHIV receives adherence counseling which aims to help them by taking control on those aspects that they do have control over—their health and (hope for) future aspirations.

Disclosure of HIV status to others outside of the family was problematic for most ALHIV in our study. Older adolescents in our study revealed that their friends were the most important sources of support and comfort. However, despite these bonds, most of them were reluctant to share their status with their friends, even in the instances where they had a desire to do so. As reported in other studies, fear of HIV-related stigma or worries that the relationship may change over time held them back.^10,23,25,26,31^ This suggests that ALHIV require a high level of mutual trust to disclose to persons outside of their immediate family. This also points to high levels of mistrust to others and the fear of discrimination if their status is advertently and inadvertently disclosed. Services and interventions targeted at ALHIV should provide them with appropriate tools or lifeskills programmes aimed at increasing their self-efficacy and confidence that they may better manage disclosure to others and the potential risks associated with it.^11,19,23,25,32^

As previously mentioned the participants in the current study reported having high levels of adherence at the time of the study, and discussed various motivators for taking their treatment. As already mentioned family support was a significant motivator for treatment adherence. Participants also mentioned their friends as their sources of social support, even when not disclosing their status to them. This suggests that ALHIV who are mentally in a good space, in other words, have positive mental well-being, were motivated to be adherent and remain in care. This positive mentality was associated with high self-esteem, hopes for the future, caring/concern for others and being healthy, which in turn motivated adherence.^23,25,32^

The most notable barriers to adherence that were reported in this study were disclosure, treatment fatigue and side effects of the medication.^10,11,23,30,31^ However, family support and treatment motivators (i.e. health/do not want to die, future aspirations etc) helped to offset these challenges. This suggests that while ALHIV may experience challenges to their adherence, the effects of these challenges may be overcome by increasing family support and a positive mentality to facilitate good adherence behavior.

## Study Limitations

Due to the qualitative nature of this study, there are several limitations. Firstly, due to the sensitive nature of this study, we recruited participants based on thoughtful considerations taken in conjunction with their health care providers. Therefore, we were only able to recruit participants who were willing to engage with the project and talk about their experiences living with HIV. Adolescents who have not been disclosed to or those who are not comfortable discussing their status were not included in the study. It may be that those who choose to hide their status have different experiences with adherence than those who were willing to share their experiences. Secondly, as this is a case study of a specific health care facility, we are not able to generalize our findings. The participants in the study represent a largely homogenous group who lived in an urban area that is characterized by a lack of resources and poverty. However, we believe that this case study provides valuable insight into the challenges HCWs face and the experiences of ALHIV accessing treatment at this facility, and similar ones in the Western Cape province. This information may be used to build on future studies aimed at improving adolescent adherence within South African public health care facilities.

## Conclusion

The findings from this case study provide valuable insights regarding the treatment experiences and services ALHIV receive at a public health care facility in the Western Cape province of South Africa. Our findings suggest that issues around disclosure remain a challenge for both HCWs and ALHIV and may present as a barrier to treatment adherence if not addressed as this may result in various mental health challenges. Additionally, treatment fatigue and side-effects were highlighted as potential barriers to adherence. On the other hand, ALHIV discussed that barriers to treatment may be overcome by various motivators to adherence which included adopting a positive mentality and perceived family and peer support. Family support was seen as instrumental in maintaining adherence as can be seen in the implementation and success of the Family Club intervention. As such, interventions aimed at improving adherence among ALHIV should incorporate elements of family support. However, such interventions should also consider the multiple contexts adolescent develop and engage in. Therefore, the intervention should also include psychosocial components to help ALHIV manage issues around disclosure and increase peer support to improve their overall mental wellbeing.
